# Characterizing Human–Dog Attachment Relationships in Foster and Shelter Environments as a Potential Mechanism for Achieving Mutual Wellbeing and Success

**DOI:** 10.3390/ani10010067

**Published:** 2019-12-30

**Authors:** Lauren E. Thielke, Monique A.R. Udell

**Affiliations:** Department of Animal & Rangeland Sciences, Oregon State University, Corvallis, OR 97331, USA; Monique.Udell@oregonstate.edu

**Keywords:** attachment behavior, shelter dog, foster dog, disinhibited attachment, attachment style

## Abstract

**Simple Summary:**

The majority of research on attachment behavior in dogs has focused on the bonds between pet dogs and their owners. In this study, we examined attachment relationships between dogs living in animal shelters and foster homes and their temporary caregivers-shelter volunteers or foster volunteers, respectively. We also examined these results in relation to previously published data from pet dogs in order to contextualize our findings. Our findings indicate that the percentage of securely attached shelter dogs was significantly lower than that previously observed in scientific studies of the pet dog population. No differences were found between proportions of securely attached foster dogs and prior research with pets. We did not find significant differences between foster and shelter dogs in terms of attachment style proportions. We also found evidence of disinhibited attachment, which is associated with a lack of appropriate social responses with unfamiliar and familiar individuals in foster and shelter dogs. This is the first study to apply attachment theory to foster and shelter settings.

**Abstract:**

This study aimed to characterize attachment relationships between humans and dogs living in animal shelters or foster homes, and to contextualize these relationships in the broader canine attachment literature. In this study, 21 pairs of foster dogs and foster volunteers and 31 pairs of shelter dogs and shelter volunteers participated. Each volunteer–dog dyad participated in a secure base test and a paired attachment test. All volunteers completed the Lexington Attachment to Pets Scale (LAPS), a survey designed to measure strength of attachment bonds as reported by humans. Although no significant differences were present in terms of proportions of insecure and secure attachments between foster and shelter populations, proportions in the shelter population were significantly lower (*p* < 0.05) than the proportions of attachment styles that would be expected in a population of pet dogs based on the published literature on pet dog attachment styles. Additionally, findings are presented in relation to data from a paired attachment test that demonstrate foster and shelter dogs spend more time in proximity to humans when the human is actively attending to the dog and encouraging interaction, as would be expected based on previous studies. We also present findings related to the presence of disinhibited attachment (previously reported in children who spent a significant portion of time living in institutionalized settings) which is characterized by a lack of preferential proximity seeking with a familiar caregiver and excessive friendliness towards strangers in foster and shelter dogs.

## 1. Introduction

Although it is widely agreed that dogs and humans form attachment relationships with one another, the method of applying attachment styles to pet dog research is a fairly recent area of interest. Previous studies have explored attachment relationships in pet dogs [1,2,3] and in the ability of shelter dogs to form attachment relationships to an unfamiliar human in a shelter setting [4]. More recently, research has shown that dogs’ behavior in attachment tests can be used to categorize dogs into attachment styles [5,6,7] originally described in literature focusing on infant–mother attachments [8]. While additional attachment styles have been described, the three primary attachment styles have commonly been defined in infant research as follows: secure (the infant shows signs of distress when separated from the mother and seeks proximity and contact when reunited), insecure-avoidant (the infant does not show much distress and does not seek proximity when reunited) and insecure-resistant (the infant is very distressed when the mother is absent but is not calmed when the mother returns and resists contact) [8].

While these attachment styles have since been applied to the pet dog–owner relationship, this component of attachment theory has not previously been applied to dogs in foster and shelter settings. Given that dogs living in animal shelters have been found to quickly form bonds to new humans [4] understanding the nature of these bonds, including the degree of attachment security that exists (which could have welfare implications) as well as similarities and differences with respect to the dog–‘owner’ bond seem especially relevant. Furthermore, although disinhibited attachment has been described among human children placed in homes after early life experiences in institutionalized settings [9], this topic has not previously been explored in dog attachment, and could be of particular interest for dogs in shelter settings. Disinhibited attachment is characterized by a lack of attenuating social responses to adults of varying familiarity, low levels of checking in with a familiar attachment figure in a stressful situation, and inclination to go off with an unfamiliar person. Disinhibited attachment can be mild or severe, and it can occur in individuals with any attachment style, although in humans, it is most pronounced in children with secure attachment styles.

Dogs that are housed in shelters for a prolonged period of time may be more likely to develop new behavior problems [10,11], or experience higher stress levels [12] and socio-cognitive declines [13]. For a review of sheltering’s effects on dog behavior, welfare and physiology, see [14]. However, regular interactions with a person have been associated with improved behavioral outcomes [15,16] and decreased cortisol levels [17,18]. Another unexplored benefit of volunteer programs in which dogs are provided with opportunities to regularly interact with familiar people may be the opportunity for transitioning dogs to develop a secure attachment bond with these volunteers, which has been found to promote positive behavioral and cognitive outcomes in both pet dogs [19] and human children [20]. Orphaned children have been shown to have a greater likelihood of thriving and developing secure social bonds later in life if they develop a secure bond with a foster parent [21].

In this study, we used the Lexington Attachment to Pets Scale (LAPS) [22] to examine levels of attachment foster and shelter volunteers report feeling towards partnered dogs. This can allow us to gain a better understanding of how volunteers perceive their relationships to dogs in these settings. In many cases volunteers are responsible for carrying out the majority of enrichment and socialization activities that dogs experience while living in an animal shelter, directly impacting the welfare of animals housed there. However, shelter staff and volunteers—especially those with animal contact—often experience burnout. Conflicted feelings about bonding with dogs under their care is one potential source of stress for these individuals, due to concerns about how the dog will feel when separated from them at the time of adoption. Shelter and foster volunteers may also miss animals they have bonded strongly with, and thus may experience feelings of loss even if they are happy a dog has been adopted. However, potential benefits and costs of the bonding experiences shared between shelter and foster volunteers and dogs in their care are not well understood.

Given that dogs living in animal shelters have been found to quickly form bonds to new humans [4], foster dogs are likely primed to form some kind of attachment to their new caretaker quickly as well. However, the style of attachment developed depends on both foster volunteer and foster dog behavior [3,23]. The existing body of prior research on children and pet dogs suggests that secure attachment formation in the foster home could be beneficial to foster dog welfare, improve behavior outcomes and increase the speed and likelihood of secure bond formation with their new owner in the adoptive home [19,20,21]. More information about how attachment bonds within the foster home are associated with foster volunteer perception, foster dog welfare and future adoption success could be used to promote optimal fostering practices that take into account both dog and volunteer wellbeing. There is also a great need to evaluate the relative benefits of fostering, including the potential for stable bonding opportunities for stray and relinquished dogs, compared with other in-shelter socialization opportunities. In many cases, it is not feasible for shelters to foster their entire canine population, and therefore there is a critical need for an empirical investigation into how regular interactions with a familiar volunteer affect shelter dog welfare and adoption outcomes. However, to date, the potential benefits of regular interactions with a familiar volunteer on dog welfare, including the potential to positively impact the formation of future bonds with adopters, has not been evaluated in shelter dogs.

The goals of this project included identifying different volunteer–dog attachment profiles using data from a behavioral test and from a scale measuring volunteer-reported attachment levels with shelter and foster dogs. We compared relative preference for an unfamiliar person in a paired attachment test, and also analyzed these data in conjunction with volunteer-reported attachment levels. In addition, we explored whether behaviors associated with disinhibited attachment in humans were also present among the foster and shelter dogs that participated in this study. Given the role of secure attachment formation in terms of positive behavioral and cognitive outcomes in human children, we wanted to explore attachment relationships in foster and shelter dogs. As shelter dogs have been shown to form attachments to unfamiliar people quickly [4], we wanted to discover whether attachment relationships between foster and shelter volunteers and dogs in these settings are secure, and to what extent they are similar to attachments seen in pet dogs living in homes. This is the first study looking at the quality of attachment using attachment styles in foster and shelter dogs.

## 2. Materials and Methods

### 2.1. Animal Subjects

Foster dog subjects included 21 dogs living in foster homes with volunteers of Willamette Humane Society in Salem, Oregon and other local rescue groups, including Senior Dog Rescue of Oregon and Greenhill Humane Society in Eugene, Oregon. Shelter dog subjects consisted of 31 shelter dogs at Willamette Humane Society. All dogs were spayed or neutered prior to participation in the study. Shelter dogs were selected by volunteers, and foster dogs were assigned to foster homes by animal shelter and foster staff. All dogs were eligible for adoption at the time of participation in the study. See Table A1 in Appendix A for a description of all subjects. All procedures were approved by Oregon State University’s institutional ethical review boards, animal related procedures were covered under OSU ACUP #4837.

### 2.2. Human Participants

Foster participants included 20 foster parent volunteers. Although all foster parent volunteers were invited to participate in a second round of testing, only one volunteer participated in a second testing session. (In some cases, testing was not possible because foster dogs were returned to the shelter before sessions could be conducted.) Shelter participants included 20 shelter volunteers that interact with dogs regularly as part of their volunteer duties. Twenty shelter-dog pairs took part in the first round of testing, and all volunteers were invited to participate in a second round of testing with a different dog. A total of 11 volunteers from round one participated in round two of testing. All analyses focusing on dog behavioral testing have been pooled. However, survey measures do not include pooled data to avoid partial dependence within the data set. Each participant provided informed written consent to participate in the study. All procedures were approved by Oregon State University’s institutional ethical review boards, human related data collection was covered under IRB #7818.

### 2.3. Behavioral Tests and Surveys

For dogs residing in foster homes, all testing was conducted at least three days after the dog entered the foster home. Participating shelter volunteers were asked to select a dog that they had interacted with for at least three separate ten-minute sessions, as this has previously been established as a sufficient amount of time for shelter dogs to establish attachment relationships with an unfamiliar person [4]. Testing sessions consisted of a Secure Base Test, designed to assess attachment relationships between dogs and familiar humans, immediately followed by a Paired Attachment Test, which aims to assess preference for a familiar vs. unfamiliar human. Tests were always conducted in this order. While both the Strange Situation Test (SST) and Secure Base Test have been validated for use with dogs, we chose to use the secure base test methodology (modeled after the first tests [24] designed to measure secure base and social preferences of this type) because it has several methodological advantages noted in the prior literature including reduced testing time, a reunion phase by the ‘caretaker’ that directly follows the alone phase, the elimination of order effects and the focus on the alone and reunion phase which have been found to produce the most reliable results in the human literature [25,26]. Following these behavioral tests, volunteers were asked to fill out a series of surveys, including the Lexington Attachment to Pets Scale.

#### 2.3.1. Secure Base Test (SBT)

All SBT sessions were conducted in a testing room unfamiliar to the dog. All testing sessions took place in a location that was unfamiliar to the dogs. In some cases, foster dogs were tested in the Oregon State Human-Animal Interaction Lab’s on-campus testing space, but most dogs were tested at the Willamette Humane Society or Greenhill Humane Society in a novel testing room. Two chairs were placed in the room, and a semi-circle of 1 m in radius was taped on the floor around the chair prior to the beginning of the testing session. Three toys of different types were placed on the floor (outside of the 1-m radius circle) before volunteers and dogs entered the testing room. Toys included a tennis ball with a squeaker, a rope toy and a stuffed toy with a squeaker.

**Phase one (Baseline, two minutes):** The familiar volunteer was asked to sit neutrally in a chair within the 1 m radius circle. The volunteers were permitted to interact freely (petting, talking, etc.) with the dog (without restraining it) each time the dog placed at least two paws in the circle, but were instructed to sit neutrally if the dog exited the circle. Dogs were able to freely explore the room. The volunteer could play with toys with the dog if the dog brought them to the volunteer inside the circle.
**Phase two (Alone, two minutes):** The familiar volunteer or adopter exited the testing room so that the dog was left alone.
**Phase three (Return, two minutes):** The familiar volunteer or adopter re-entered the testing room and the instructions were identical to phase 1 (baseline).

#### 2.3.2. Video Analysis of SBT

Two independent coders reviewed the return phase videos for each dog’s SBT and categorize dogs’ attachment styles based on patterns of behavior seen in the return phase. A holistic analysis was used for these categorizations (see [5,6,7]). Inter-rater reliability was assessed based on the percentage of independent agreement after this initial round of coding. After the two coders reviewed each video independently, they watched any videos for which they disagreed on attachment style categorization together and reached an agreement. A description of all attachment style classifications can be found in Table 1. Degree of independent agreement among coders for attachment style was 72%, and a consensus was reached for all dogs when coders reviewed videos together.

#### 2.3.3. Paired Attachment Test

The Paired Attachment test included the following phases:

**Phase 1 (two minutes):** A two-minute alone period immediately following the SBT.
**Phase 2 (Passive, two-minute phase)**: The dog’s caretaker and a stranger sat neutrally for two minutes in chairs opposite each other surrounded by a 1m radius circle. Each individual was instructed to pet the dog twice each time it entered the circle with at least two paws, but were instructed to otherwise remain neutral.
**Phase 3 (Active, two minutes):** Both humans were asked to call the dog and provide continuous petting and attention if the dog entered their circle with at least two paws.

**Video analysis of Paired Attachment Test**

**Figure 1:**
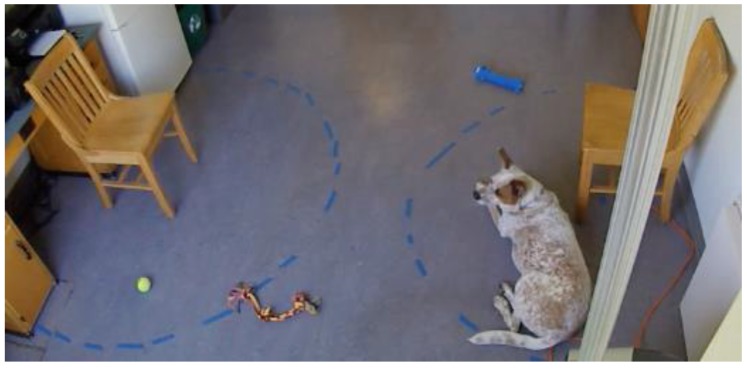
Paired attachment test set-up.

All videos were coded across phases for first person approached (unfamiliar or familiar), duration of proximity seeking with each person, and duration of contact with each person. An ethogram can be found in Table 2. Please see Figure 1 for a picture of the Paired Attachment test set-up.

#### 2.3.4. Disinhibited Attachment Coding

Based on the methods used in [9], we developed a scale to assess disinhibited attachment using rankings of different measures that were combined into a composite score. We assigned rankings to each dog based on the proportion of the return phase of the SBT spent seeking proximity to the familiar person. Because severe disinhibited attachment is associated with a lack of proximity seeking with a familiar person, the highest proportion of time spent in proximity to the familiar person received the lowest rankings. Inter-rater reliability for this measure was 75%. In addition, we assigned rankings based on total amount of time spent in proximity (within the 1-m radius circle taped on the floor around the chair) to the unfamiliar person across both phases of the Paired Attachment Test. Inter-rater reliability for total proportion of time spent in proximity to the unfamiliar person was 93.8%. Low proximity seeking with the unfamiliar person received low rankings on the scale; higher proportions of time spent proximity seeking received higher rankings. Across all measures, in the case of a tie, those values were assigned the same rank. For instance, if two dogs spent 98.3% of the session seeking proximity to the familiar person, they would receive the same rank. Both scores were summed to create an overall disinhibition score.

### 2.4. Statistical Methods

All statistical analyses were conducted using R Studio (version 1.1.463). All statistics were two-tailed with an alpha level of *p* < 0.05.

#### 2.4.1. Attachment Analysis

A Fisher’s Exact Test was used to compare proportions of securely and insecurely attached dogs within foster and shelter groups. A Chi Square test was used to compare the proportions of attachment styles seen in the foster and shelter populations to expected frequencies of proportions of pet dogs based on all published literature categorizing attachment styles in pet dogs [5,6,7]. A McNemar’s Test was used to compare dogs’ attachment styles for shelter volunteers who participated in two separate rounds of testing.

#### 2.4.2. Paired Attachment Analysis

Normality was assessed using the Shapiro–Wilk Test. For shelter and foster groups, all proximity and contact data were not normally distributed (*p* < 0.05). For both foster and shelter groups, a Kruskal–Wallis test was used to assess whether differences in proximity or contact seeking were present with respect to whether the humans were passively interacting with the dogs (two pets every time the dog enters each person’s respective circle) or actively encouraging interaction from the dogs. Post hoc comparisons were made using Wilcoxon Signed-Rank Tests for within-subject comparisons across phases. Fisher’s Exact tests were used to determine whether the first person approached (unfamiliar or familiar) varied according to dog source (shelter or foster) or attachment style (insecure or secure) for active and passive phases. Mann–Whitney *U*-tests were used to compare total amount of time spent with the familiar person vs. the unfamiliar person across both passive and active phases for both populations (shelter and foster) and attachment style (secure vs. insecure). A difference score was calculated to assess overall preference for a familiar human vs. an unfamiliar human by subtracting the total proportion of time spent with an unfamiliar person across active and passive phases from the total proportion of time spent with a familiar person across active and passive phases, and these data were not normal, *p* < 0.05. See Table 3 for inter-rater reliability scores for all measures. Percent agreement between two coders was assessed using an 8% tolerance.

#### 2.4.3. Disinhibited Attachment Analysis

Normality of disinhibited attachment scores was assessed using the Shapiro–Wilk test, *p* > 0.05. *T*-tests were used to compare disinhibition scores across four categories: insecure shelter dogs, secure shelter dogs, insecure foster dogs, and secure foster dogs. We also used Mann–Whitney *U*-tests for non-normally distributed data for proportion of time spent in proximity to familiar person for analyses by group and by attachment style.

#### 2.4.4. Lexington Attachment to Pets Scale (LAPS) Analysis

Normality was assessed using the Shapiro–Wilk test and LAPS data were normal, *p* > 0.05. Because assumptions of normality were not violated, parametric statistics were used to compare between groups. A two-way ANOVA with attachment category and environment (foster vs. shelter) and possible interactions was used to analyze LAPS data. A Pearson’s Correlation was used to determine whether there was a relationship between LAPS score and overall preference score for a familiar vs. unfamiliar person. Only data from the first round of shelter and foster volunteer participation were used for these comparisons, in order to avoid any confounding effects of volunteers who participated in both rounds. A Pearson’s Correlation was used to assess whether scores from shelter volunteers’ first participation were related to LAPS scores from shelter volunteers’ second participation.

## 3. Results

### 3.1. Attachment

**Figure 2:**
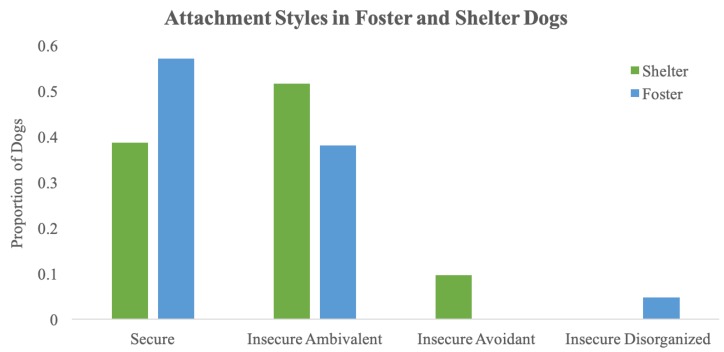
Proportion of dogs categorized into each attachment style for foster and shelter dog populations.

Within the foster group, a total of twelve dogs (57.14%) were categorized as secure and nine dogs (42.86%) were categorized as insecure (eight dogs (38.10%) were categorized as insecure-ambivalent, and one dog (4.76%) was categorized as insecure-disorganized). Within the shelter group, a total of twelve dogs (38.71%) were categorized as secure and a total of nineteen dogs (61.29%) were categorized as insecure (sixteen shelter dogs were scored as insecure ambivalent (51.61%), and three insecure shelter dogs (9.68%) were scored as insecure avoidant). The Fisher’s Exact test comparing the proportion of insecure and secure dogs in the shelter and foster groups was not significant (*p* = 0.26). See Figure 2 for a comparison of attachment styles in each population.

**Figure 3:**
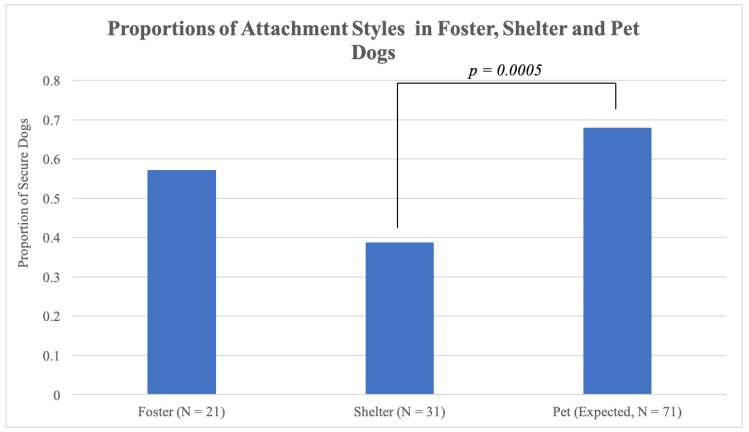
Proportions of observed foster and shelter dogs categorized as secure, compared to expected proportions of pet dogs with secure attachments based on published literature [5,6,7].

To obtain an overall picture of how each population of foster and shelter dogs, respectively, compared to pet dogs in terms of proportions of attachment styles, we summed data from all published literature involving categorization of attachment styles in pet dogs for each category of attachment styles (secure, insecure-ambivalent, insecure-avoidant, and insecure-disorganized) [5,6,7]. Only data from dogs in the saline condition were used for [6], a study which included a counterbalanced repeated measures design in which oxytocin was administered during one testing session and saline upon another visit prior to participating in the secure base test, to avoid dependence (i.e., if we did not exclude the oxytocin sessions, we would be using data for the same dogs twice) and any effect of oxytocin on behavior. Across previously published studies, 68% of pet dogs had been categorized as having a secure attachment to their primary caretaker and 32% of pet dogs had been categorized as displaying a type of insecure attachment. The proportion of secure shelter dogs significantly differed from what was expected when compared against previously published attachment outcomes in pet dogs, χ^2^ (1, *N* = 31) = 12.22, *p* = 0.0005 (Figure 3). No significant differences were found when proportions of attachment styles for the foster dog group were compared to pet dog attachment style proportions, *p* > 0.05 (Figure 3).

No significant differences were found in terms of comparisons of attachment styles among dog–volunteer dyads for shelter volunteers who participated in two rounds of testing with two different dogs, *p* = 0.62.

### 3.2. Paired Attachment

A trend was found for proportion of time shelter dogs spent seeking proximity to both the familiar and unfamiliar person when all conditions (familiar passive, familiar active, unfamiliar passive, unfamiliar active) were compared to each other, H (3) = 7.80, *p* = 0.05. A significant difference was found with respect to the proportion of time spent in contact across all conditions, H (3) = 23.103, *p* = 3.84 × 10^−5^. This difference was driven by the finding that shelter dogs spent significantly more time in contact with both the familiar person (W = 456, *p* < 0.001) and the unfamiliar person (W = 68, *p* = 0.001) in the active phase of the sociability test when compared to the passive phase. No significant differences were found in terms of proportion of time spent in contact with the unfamiliar person vs. the familiar person (*p* > 0.05 for both the passive and active phases).

For the foster group, a significant effect was found with respect to the proportion of time spent in proximity across all conditions, H (3) = 11.49, *p* = 0.01. A trend was present with respect to the proportion of time foster dogs spent in proximity to the familiar person compared to the unfamiliar person in the active phase, W = 165, *p* = 0.09. The median proportion of time foster dogs spent with the familiar person in the active phase was 0.52, and the median proportion of time spent in proximity to the unfamiliar person in the active phase was 0.28. A trend was also found within the foster group with respect to proportion of time spent in contact H (3) = 9.23, *p* = 0.03. After Bonferroni correction, the significance threshold for the Kruskal–Wallis test was 0.0257.

Based on the overall score for time spent with the familiar person vs. time spent with the unfamiliar person across phases, no significant differences were found with respect to group (foster vs. shelter) or attachment style (secure vs. insecure). In addition, we analyzed whether differences were present with respect to overall time spent in proximity to the familiar person compared to the unfamiliar person, and no significant differences were found with respect to group or attachment style, *p* > 0.05. No significant differences were found with respect to first person approached for active or passive phases when shelter and foster dogs were compared across groups, *p* > 0.05.

### 3.3. Disinhibited Attachment

**Figure 4:**
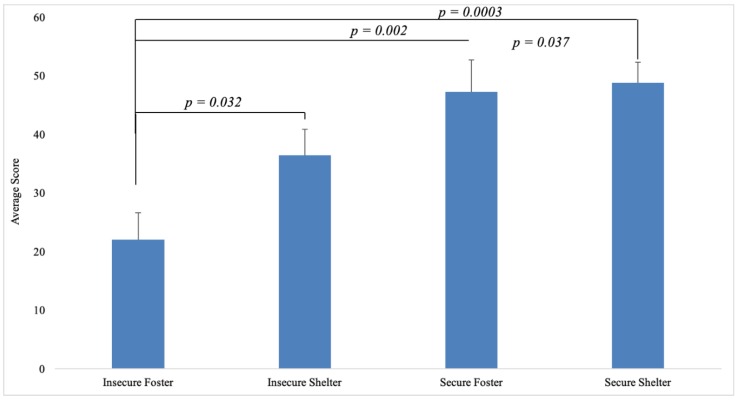
Mean disinhibited attachment scores for foster and shelter dogs with insecure and secure attachment styles. Error bars indicate standard error of the mean.

Insecure foster dogs had the lowest disinhibited attachment rankings, with a mean of 22.0. Insecure shelter dogs had the second lowest scores on average with a mean score of 36.42. The mean score for the secure foster group was 47.17 and the mean score for the secure shelter group was 48.75. Insecure foster dogs displayed significantly lower mean disinhibition scores than secure foster dogs, *t*(18.99) = −3.5499, *p* = 0.002. Insecure foster dogs also displayed significantly lower mean scores of disinhibited attachment compared to secure shelter dogs, *t*(16.33) = −4.64, *p* = 0.0003. Furthermore, insecure foster dogs scored significantly lower on disinhibition than insecure shelter dogs, *t*(21.57) = −2.2835, *p* = 0.03. In addition, insecure shelter dogs displayed significantly lower disinhibited attachment scores than secure shelter dogs, *t*(28.97) = −2.184, *p* = 0.04. No significant differences were present with respect to insecure shelter dogs and secure foster dogs, *p* > 0.05. In addition, we did not find significant differences between secure foster dogs and secure shelter dogs on disinhibition, *p* > 0.05. See Figure 4 for mean scores on disinhibited attachment among foster and shelter dogs with secure and insecure attachments.

### 3.4. Lexington Attachment to Pets Scale (LAPS)

In terms of the results of the two-way ANOVA analysis of LAPS scores, no significant main effect or interaction effect was found, F (1, 36) = 0.20, *p* > 0.05 for attachment style categorization or group (shelter/foster). The average LAPS score for shelter volunteers was 15.45 and the average LAPS score for foster volunteers was 20.40. This difference was not statistically significant, *t*(37.96) = 1.11, *p* > 0.05. The average LAPS score for insecure dogs was 18.60 and the average LAPS score for secure dogs was 17.25. This difference was not statistically significant, *t*(37.16) = 1.11, *p* > 0.05. Based on comparisons between first participation and second participation for the 11 volunteers that participated twice, there was a positive correlation between LAPS scores on each round, r = 0.62, *p* = 0.04. With respect to the familiar person in the active phase, there was a significant positive correlation between the amount of time spent proximity seeking and the LAPS score, r = 0.35, *p* = 0.03. There was not a significant correlation between LAPS scores and the amount of time spent seeking proximity with the familiar person in the passive phase, *p* > 0.05.

**Figure 5:**
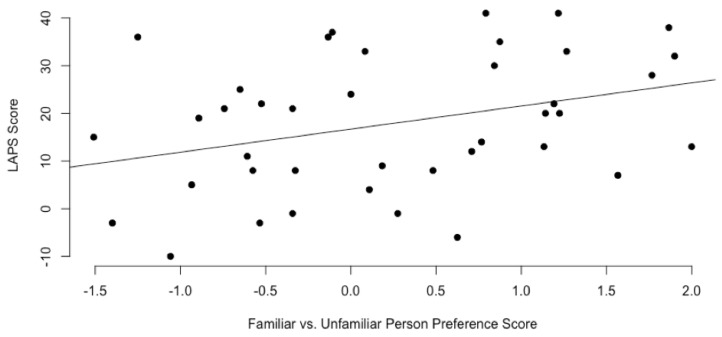
Scatterplot for overall score for preference for a familiar human compared to an unfamiliar human compared to LAPS scores. Positive preference scores indicate a preference for the familiar human; negative preference scores indicate a preference for the unfamiliar human. Higher LAPS scores indicate a stronger degree of attachment; lower LAPS scores indicate a weaker degree of attachment, as reported by familiar volunteers. This suggests that there is a relationship between the strength of attachment human volunteers feel for a dog and the amount of preferential proximity seeking that dog displays in an attachment, however the direction and causality of the relationship is unknown.

There was a significant positive correlation between a dog’s preference score (calculated by subtracting the total proportion of time spent with an unfamiliar person across active and passive phases from the total proportion of time spent with a familiar person across active and passive phases) for the familiar person compared to an unfamiliar person on the Paired Attachment Test and the strength of attachment reported by the caretaker/familiar person on the LAPS survey, r = 0.34, *p* = 0.03 (Figure 5).

## 4. Discussion

To our knowledge, this is the first application of attachment style categorization in human–dog relationships in foster and shelter settings. We explored the proportions of attachment relationships seen among foster and shelter volunteers and dogs in foster and shelter settings, and also explored disinhibited attachment in these populations. Our findings indicate that the proportion of secure attachment styles in shelter dogs included in this study were significantly lower than the proportion of secure attachment styles previously reported for pet dogs. Conversely, foster dogs formed secure attachments to their caretakers at rates more similar to those reported in dog–owner relationships, although no significant differences were found between proportions of attachment styles when comparing between foster and shelter dogs directly [5,6,7].

The finding that shelter dogs are significantly more likely than pet dogs (based on data from previously published literature) to form insecure attachments to their temporary caretakers is of note, especially given the high rate of insecure ambivalent attachments observed. This attachment style is associated with excessive proximity seeking upon return, even though this contact is less effective at reducing stress than in secure attachment relationships. Given that previous research has shown that lying in proximity to adopters positively influenced dog adopter decisions in a shelter setting [27], it is possible that attachment styles aligned with greater proximity seeking could have some benefit in this environment. For example, dogs behaving ambivalently might be perceived as more social and therefore be more attractive to potential adopters. However, more research should be done before conclusions can be drawn. Future research could explore whether attachment styles formed with familiar volunteers in the SBT corresponds to behavior in adopter-dog interactions at the shelter. Future studies could also explore attachment relationships between dogs that have been rehomed from foster and shelter settings and their adoptive owners, as this has not been previously explored.

We also found evidence of disinhibited attachment among dogs in foster homes and shelter settings. We expected to see higher levels of disinhibition among dogs displaying secure attachments within these settings (as that would be consistent with the human literature), and indeed, the highest levels of disinhibition were exhibited among secure shelter and foster dogs and the lowest levels among insecure shelter and foster dogs. Previous work has shown that disinhibited attachment was also present in human orphans from Romanian orphanages who had experienced institutional deprivation and children adopted in the UK who had no exposure to institutional deprivation [9]. Thus, disinhibited attachment could be a product of the temporary nature of relationships with caregivers, traumatic experiences early in life associated with becoming an orphan, or other factors beyond the quality of the environment. Shelter and foster dogs with secure attachments may show higher levels of disinhibition because it allows for greater social flexibility within these environments, which could provide advantages in terms of greater likelihood of being taken for walks, greater chances of interaction with potential adopters, or more social attention from volunteers. Experiences prior to placement in a foster home or a shelter could also contribute to the development of disinhibited attachment, and further research is needed in this area to better understand the development and implications of disinhibited attachment in foster and shelter dogs. It should also be noted that the dogs in the shelter environment receive a great deal of enrichment and social interaction, including play groups with conspecifics, training opportunities, and several walks each day. Future studies should assess disinhibited attachment in kenneled dogs that do not receive as many opportunities for enrichment and social exposure to both humans and conspecifics. We also found that insecure dogs spent a greater proportion of time in proximity to the familiar person during the SBT than secure dogs. Although disinhibited attachment has not previously been reported in canine attachment literature, it appears to be relevant for dogs in shelter and foster settings. To date no research has been done on disinhibition in pet dogs, so it is unknown to what degree these results may differ from pet dog populations, but such comparisons merit further investigation. Furthermore, additional aspects of disinhibition could be explored. For example, in the current study, we did not look at the dog’s willingness to leave with the stranger. This would be an interesting additional condition for future studies, since it is one trait often noted in human children exhibiting disinhibited attachment disorders.

The results of the paired attachment test suggest that shelter dogs are more likely to seek contact depending on attentional state (i.e., whether the person is actively interacting with the dog/encouraging attention or sitting passively and petting the dog twice each time it enters the 1m radius circle surrounding the person’s chair), while foster dogs are more likely to seek proximity regardless of whether the familiar volunteer or unfamiliar person are actively attending to the dog. We found that shelter dogs were attentive to the attentional state of the person to a greater degree than foster dogs. It is possible that although foster homes may allow for an easier transition to an adoptive home environment, that dogs living in foster homes may be less attuned to human attentional state. Shelter dogs, on the other hand, typically spend more time in social isolation than foster dogs, and paying close attention to the attentional state of humans may provide benefits, such as increased socialization, exercise, or interactions with adopters. Furthermore, no significant differences were found with respect to contact or proximity seeking when comparisons were made based on attachment style. It should also be noted that no differences were found with respect to the first person approached by either shelter or foster dogs. Overall, no differences were found between foster and shelter dogs based on preference for an unfamiliar or a familiar person in the paired attachment test. We did not find any significant differences in terms of attachment style and preference for an unfamiliar person compared to a familiar person. This indicates that both shelter and foster dogs show flexibility in terms of social interaction.

We found a significant correlation between LAPS scores, which measured the attachment strength of shelter and foster human volunteers, and a dog’s preference for that volunteer versus an unfamiliar human in the Paired Attachment test. This indicates that familiar volunteers whose dogs exhibited a stronger preference for them (i.e., dogs who spent more time in proximity to the familiar person across phases), also reported a stronger attachment to their chosen dog (or vice versa). While the causality of this relationship cannot be determined from the current data, this connection warrants further investigation in the future, although regardless of causality, this finding is interesting as it indicates reciprocity in the attachment relationship.

The reported strength of attachment by volunteers (LAPS scores) did not differ between shelter and foster volunteers. Although we expected that foster volunteers would report a greater degree of attachment to foster dogs than shelter volunteers to shelter dogs, this was not the case. Thus, it is possible that they were primed to think of themselves as having a stronger attachment to the dog that they chose for the study than they would have naturally. Future studies could examine LAPS ratings for shelter volunteers more generally, to determine if the level of attachment reported towards dogs in this study was unique (and possibly due to the experimental context) or if shelter volunteers commonly feel highly bonded to shelter dogs. Another possibility is that foster volunteers may try to avoid forming strong attachment bonds with foster dogs, to protect their own emotions or to protect the dog from perceived feelings of loss when eventually rehomed. Such strategies might also be used to reduce temptation to adopt the foster dog. Future research could measure LAPS scores in relation to several foster dogs, including foster volunteers who did and did not adopt their foster dogs, in order to determine whether strength of attachment, as reported by the person, plays a role in decision-making regarding adoption of foster dogs by their foster parent volunteers. It is also of note that the correlation between LAPS scores and proximity seeking towards volunteers appears to be driven by the active phase of the test, where the volunteer can call and interact with the dog. This suggests that the attachment strength and behavior of the familiar person may be influencing the dogs’ attachment behavior, but more studies are needed to be sure. While future research should directly evaluate long term outcomes of foster and shelter dogs who formed secure and insecure attachment bonds to transitional caretakers, in the human literature, orphaned children who develop secure bonds to a foster parent are more likely to form secure social bonds later in life [20]. If this proves to be true of dogs as well, this would suggest that fostering may provide an additional benefit by increasing the likelihood of a dog establishing a secure attachment prior to final adoption.

It should be noted that differences were not found with respect to attachment styles of dogs for volunteers who participated in the first and second round of testing. However, only eleven shelter volunteers participated in the first and second round of testing, resulting in a relatively small sample size. For six of eleven participants, the dogs that they participated in the SBT with in the first round were insecurely attached. Based on these data, we cannot conclude whether the attachment styles dogs presented with in the SBT were due to the nature of their interactions with the volunteers, or due to personality traits of the dogs, or a combination of these factors. It would also be interesting to evaluate whether volunteer personality plays a role in dog selection. For instance, do volunteers with certain personality traits, such as higher anxiety levels, choose more anxious dogs? Research with owners of pet dogs has shown that attachment avoidance in people on the Adult Attachment Scale is associated with increased occurrence of separation anxiety in their dogs [28].

Another finding with respect to LAPS scores was that no differences were present with respect to attachment security. It is possible that volunteers are not attuned to attachment style-related behaviors, and therefore evaluate attachment to shelter and foster dogs based on other observations. It is also possible that some volunteers prefer dogs who are securely attached and others prefer dogs who are insecurely attached. Future research could evaluate the attachment styles of dogs owned by volunteers in conjunction with SBT results between volunteers and dogs in foster and shelter settings, in order to gain a better understanding of the role of the human in forming and maintaining attachment relationships in these contexts. Further research is needed to determine whether LAPS scores correspond to attachment style. Also, of note is that even with a relatively small sample size of 11 volunteers who participated in both rounds of testing in the shelter setting, there was a trend of a significant correlation between LAPS scores in each round. This suggests that volunteer personality and individual framework for attachment relationships may play a role in the formation and maintenance of social bonds between volunteers and dogs in animal shelters. It is also important to note that volunteers have many opportunities to interact with a variety of dogs, as dogs are typically adopted out relatively quickly from the shelter at which this study was conducted, and thus, it is remarkable that volunteers form strong attachment bonds to dogs over a relatively short time period. It is also possible that volunteers with lower LAPS scores may attempt to avoid developing attachment bonds to individual dogs at the shelter, and this could be a coping mechanism to help prevent compassion fatigue.

## 5. Conclusions

We have shown that shelter dogs differ significantly from a meta-analysis of pet dogs with respect to proportions of attachment styles, while no differences were found between attachment proportions of shelter and foster dogs. This provides evidence that social relationships formed between foster parent volunteers and foster dogs in the foster home may be more similar in nature to those formed in the typical home environment of pet dogs. Therefore, fostering dogs may provide an important additional benefit, by increasing the likelihood that a dog will experience the establishment of a secure attachment to a caretaker before final adoption. In studies with human children, this scenario not only allowed for better coping with stress in the short term, but also increased the likelihood that the child would develop a secure attachment to their caretaker in their final home upon being adopted. More research is needed to evaluate if the same is true for adopted foster dogs. Additionally, disinhibited attachment, characterized by excessive friendliness towards unfamiliar people, lack of discrimination among adults based on social familiarity, and lack of checking in with an attachment figure during a stressful situation in humans, is present in shelter and foster dogs. We also found preliminary evidence that would suggest a relationship between how attached a human volunteer feels towards a dog in their care and the dog’s behavior towards that person, however more research is needed to determine if a causal relationship exists, and if so, the direction of that relationship.

## Figures and Tables

**Table 1:** Holistic coding attachment style definitions (adapted from [5]).

Attachment Style	Definition
**Secure**	Little or no resistance to contact or interaction. Greeting behavior is active, open and positive. Seeks proximity and is comforted upon reunion, returning to exploration or play.
**Insecure ambivalent**	Shows exaggerated proximity-seeking and clinging behavior, but may struggle if held by familiar volunteer. Mixed persistent distress with efforts to maintain physical contact and/or physically intrusive behavior directed toward the familiar volunteer. (Dogs who the judges agreed seemed essentially secure but with insecure ambivalent tendencies, were included in the secure group).
**Insecure avoidant**	May show little/no distress on departure. Little/no visible response to return, ignores/turns away but may not resist interaction altogether (e.g., rests or stands without bodily contact, out of reach or at a distance).
**Insecure disorganized**	Evidence of strong approach avoidance conflict or fear on reunion, for example, circling familiar volunteer, hiding from sight, rapidly dashing away on reunion, “aimless” wandering around the room. May show stereotypies on return (e.g., freezing or compulsive grooming). Lack of coherent strategy shown by contradictory behavior. “Dissociation” may be observed, that is, staring into space without apparent cause; still or frozen posture for at least 20 s (in the nonresting, nonsleeping dog).

**Table 2:** Paired Attachment ethogram.

Behavior	Definition
**Proximity seeking**	Proportion of the episode in which the dog had at least 2 paws (or half their body) within the 1 m radius circle the human was sitting in.
**Dog-human contact**	Proportion of the episode in which the dog or human engaged in physical contact with the other individual. Contact must be in circle to count. Sniffing and body touches count as contact.

**Table 3:** Inter-rater reliability for all Paired Attachment measures.

Measure	Percent Agreement between Two Coders
Familiar human: proximity seeking (duration)	95.3%
Familiar human: contact (duration)	87.5%
Unfamiliar human: proximity seeking (duration)	100%
Unfamiliar human: contact (duration)	92.2%

## Appendix A

**Table A1:** Subjects’ demographic information, including names, groups, ages, modes of intake, length of stay (LOS), and breed (as listed by their associated shelter or rescue group). Several of the dogs in the study were returned; length of stay was calculated based on initial intake date and final adoption date. All dogs were spayed and neutered prior to participating in the study. Length of stay data were not available for some dogs that participated, and in a few cases (noted below), dogs were still available for adoption at the completion of the study. All breed determinations were based on visual inspection by the participating shelters and rescue groups, and it is important to note that reliability for this method is low [29].

Dog’s Name	Group	Age	Mode of Intake	LOS	Breed
Whisper	Foster	8 years	Transfer	32	Mixed breed, medium
Tilly	Foster	5 years	Transfer	46	Mixed breed, small
Jazzy	Foster	1 year	Unknown	Unknown	Greyhound
Jupiter	Foster	2 years	Transfer	16	Mixed breed, medium
Maddie	Foster	4 months	Surrender	43	Mixed breed, small
Opal	Foster	2 years	Transfer	154	Mixed breed, medium
Ginger	Foster	2 years	Transfer	57	Mixed breed, medium
Hera	Foster	5 years	Transfer	70	Mixed breed, medium
Helios	Foster	2 months	Transfer	62	Mixed breed, medium
Mackenna	Foster	Unknown	Unknown	Unknown	Mixed breed, medium
Sierra	Foster	Unknown	Unknown	Unknown	Mixed breed, medium
Brisa	Foster	8 years	Surrender	Unknown	Mixed breed, large
Skipper	Foster	5 months	Transfer	52	Mixed breed, medium
Remy	Foster	1 year	Unknown	120+ (still available for adoption)	Mixed breed, medium
Lux	Foster	2 years	Unknown	Unknown	Greyhound
Lillie	Foster	13 years	Unknown	Unknown	Mixed breed, small
Dr. Zeuss	Foster	4 years	Stray	107	Mixed breed, large
Panda	Foster	4 years	Unknown	Unknown (still available)	Mixed breed, medium
Bella	Foster	8 years	Unknown	Unknown (still available)	Pomeranian
Nellie	Foster	9 years	Stray	23	Shih Tzu
Stormy	Foster	Unknown	Unknown	unknown	Mixed breed, medium
Bandit	Shelter	2 years	Surrender	87	Mixed breed, large
Biscay	Shelter	2 years	Transfer	147	Mixed breed, medium
Brownie	Shelter	5 years	Stray	30	Mixed breed, large
Charlie	Shelter	5 years	Surrender	134	Mixed breed, large
Cheyenne	Shelter	8 months	Transfer	8	Mixed breed, medium
Chico	Shelter	5 years	Surrender	31	Mixed breed, large
Clooney	Shelter	1.5 years	Stray	19	Mixed breed, large
Dakota	Shelter	5 years	Surrender	83	Mixed breed, large
Floki	Shelter	1 year	Surrender	69	Mixed breed, large
Gemma	Shelter	2 years	Surrender	35	Mixed breed, large
Hoagie	Shelter	3.5 years	Transfer	427	Mixed breed, large
Hunny	Shelter	7 years	Surrender	32	Mixed breed, small
Jordy	Shelter	1 year	Transfer	27	Mixed breed, small
Luna	Shelter	3 years	Surrender	49	Mixed breed, large
Maizie	Shelter	5 years	Transfer	6	Mixed breed, small
Lincoln	Shelter	1 year	Transfer	200	Mixed breed, large
Mari	Shelter	2 years	Surrender	159	Mixed breed, large
Numair	Shelter	7 years	Surrender	64	Mixed breed, small
Smallz	Shelter	10 years	Transfer	23	Mixed breed, small
Jacob	Shelter	6.5 years	Surrender	48	Mixed breed, small
Elfie	Shelter	4 years	Surrender	141	Mixed breed, large
Champ	Shelter	5 years	Surrender	85	Mixed breed, large
Daisy	Shelter	7 years	Surrender	39	Mixed breed, medium
Molly	Shelter	3 years	Surrender	69	Mixed breed, medium
Hans	Shelter	3.5 years	Surrender	40	Mixed breed, large
Heidi	Shelter	1.5 years	Transfer	126	Mixed breed, large
Charlie Boy	Shelter	3.5 years	Surrender	57	Mixed breed, small
Brinx	Shelter	4.5 years	Surrender	177	Mixed breed, medium
Peaches	Shelter	1.5 years	Surrender	38	Mixed breed, large
Carly	Shelter	4 years	Transfer	200	Retriever mix
Starlord	Shelter	1 year	Stray	39	Mixed breed, small
